# Qinglong Zhidong Decoction Alleviated Tourette Syndrome in Mice *via* Modulating the Level of Neurotransmitters and the Composition of Gut Microbiota

**DOI:** 10.3389/fphar.2022.819872

**Published:** 2022-03-22

**Authors:** Na Wang, Xinchen Wu, Qi Yang, Dingyue Wang, Zhao Wu, Yuanyuan Wei, Jieqiong Cui, Li Hong, Lei Xiong, Dongdong Qin

**Affiliations:** ^1^ Yunnan University of Chinese Medicine, Kunming, China; ^2^ Huanghe S & T University, Zhengzhou, China; ^3^ Department of Integrative Medicine, Huashan Hospital, Fudan University, Shanghai, China

**Keywords:** Tourette syndrome, Qinglong Zhidong decoction, gut microbiota, neurotransmitter, DA

## Abstract

Qinglong Zhidong Decoction (QLZDD), a traditional Chinese medicine (TCM) prescription, has been effectively used to alleviate Tourette syndrome (TS) in children. However, the therapeutic mechanism of QLZDD on TS has not been evaluated. The present study aims to elucidate the therapeutic effect and the possible therapeutic mechanism of QLZDD on TS in mouse model. A 3,3-iminodipropionitrile (IDPN, 350 mg/kg)-induced-TS mouse model was established. The mice were randomly divided into the control group, the model group, the haloperidol group (14 mg/kg), the low-, middle-, or high-QLZDD-dose groups (6.83 g/kg, 13.65 g/kg, 27.3 g/kg). QLZDD was administrated orally once a day for 4 weeks. The tic-like behavior was recorded weekly. Then, neurotransmitters and neurotransmitter receptors were analyzed by ELISA*,* immunohistochemistry (IHC), and quantitative reverse transcription PCR in striatum. Further, the alteration to intestinal flora was monitored by 16s rRNA sequencing, and the role of gut microbiota in the alleviation of TS by QLZDD was investigated. QLZDD ameliorated the tic-like behavior, and decreased the level of excitatory neurotransmitters such as Glu and DA and increased the level of the inhibitory neurotransmitter GABA significantly. Moreover, QLZDD significantly blocked the mRNA expression and the protein expression of D1R and D2R in the striatum, while activated the levels of DAT and GABAR. Interestingly, QLZDD mediated the composition of gut microbiota by increasing the abundance of *Lactobacillus* and *Bacteroides* but decreasing the abundance of *Alloprevotella* and *Akkermansia*. Taken together, QLZDD ameliorated the tic-like behavior in TS mouse, its mechanism of action may be associated with restoring the balance of gut microbiota and neurotransmitters. The study indicated a promising role of QLZDD in alleviating TS and a therapeutic strategy for fighting TS in clinical settings.

## Introduction

Tourette syndrome (TS) is a neurobehavioral and neuropsychiatric disorder that occurs in childhood and is characterized by repeated involuntary movements and vocal convulsions (Efron and Dale, 2018; Singer, 2019; Fernández de la Cruz and Mataix-Cols, 2020). It is estimated that the prevalence rate of TS in children and adolescents is about 1% (Robertson et al., 2017). And tic is a typical symptom of TS, which is often accompanied by other behavioral changes and mental disorders, such as attention deficit hyperactivity disorder (ADHD), obsessive-compulsive disorder (OCD), autism spectrum disorder (ASD), anxiety, depression and other neuropsychiatric disorders (Muth, 2017). Tic often impairs the quality of life of the children with TS and is accompanied by the different degree of learning disabilities (Burd et al., 2005). The etiology and the pathogenesis of TS are complex and are not yet clear.

Previous studies have shown that neuroanatomy and neurochemistry are closely related to TS. A large amount of research has found morphological and functional changes in the cortex-striatum-thalamus-cortex (CSTC) circuit and the dysfunction in the dopaminergic system of the children with TS (Singer et al., 1993; Peterson et al., 1998; Bronfeld et al., 2013). The dysfunction in the dopaminergic system is considered as the main cause of the neuropathology of TS (Singer et al., 2002). Studies have demonstrated that the abnormal activity of neurons in the striatum of the children with TS, the reduction in the volume of the caudate nucleus, and the activation of dopamine D1 (D1R) and dopamine D2 (D2R) receptors in the striatal output pathway resulting in excessive dopaminergic activity, which can enhance the motor activity of the thalamus-cortex (Wong et al., 2008). Dopamine transporter (DAT) is an important channel for regulating the concentration of dopamine (DA) in the striatum. The glycoprotein that locates in the presynaptic membrane of dopamine neurons regulates extra-synaptic DA concentration by re-ingesting DA into the neurons.

Moreover, the metabolic imbalance between the inhibitory neurotransmitter γ-aminobutyric acid (GABA) and the excitatory neurotransmitter glutamate (Glu) performs a critical function in the pathogenesis of TS. For example, by magnetic resonance spectroscopy and positron emission tomography, researchers have confirmed the decrease in GABA for the children with TS (Puts et al., 2015) and the significant increase in Glu for the patients with TS (Puts et al., 2015).

The modification of the composition of gut microbiota is associated with a variety of neuropsychiatric diseases, and microflora can regulate brain development, function, and behavior by regulating a variety of systems such as the immune system, the endocrine system, and the nervous system (Martino et al., 2018). Gut microbiota has been implicated in different mental disorders, including depression, ASD, and Parkinson’s disease (PD) (Hulme et al., 2020). It is reported that microorganisms secrete neurotransmitters (e.g., GABA), serotonin, and metabolites (e.g., short-chain fatty acids). With the stimulation of the secretions, the vagus nerve is enabled and the signal molecules that control brain activity are bound (Cenit et al., 2017). As shown, transplanting the severe TS children with the healthy children’s feces could significantly ameliorate the tic behavior (Davidson et al., 2018). These factors may relate to the progression of TS *via* the modulation of gut microbiota and neurotransmitters.

At present, the treatment agents of TS mainly include α-2 receptor agonists (clonidine and guanidine) and DA receptor antagonists (haloperidol, pimozide, and risperidone) (Zhao et al., 2017). These drugs alleviate the tic behavior; however, impose adverse reactions (Pringsheim et al., 2019b; Lewin et al., 2014). Therefore, finding suitable anti-TS drugs is still a major challenge (Zhang et al., 2016; Pringsheim et al., 2019a). The traditional Chinese medicine (TCM) can effectively improve the clinical symptoms of TS, and there is no obvious side effect (Yu et al., 2020; Zhao et al., 2020). Orally-administered TCM may balance intestinal flora and may regulate neurotransmitters for the treatment of neurological diseases such as depression, autism, and epilepsy (Mitchell et al., 2006).

Qinglong Zhidong decoction (QLZDD) (patent no. CN202010465129.5), is a prescribed Chinese herbal formula that is based on the TCM theory. The formula includes *Cynanchum otophyllum* C.K. Schneid*, Gentiana scabra* Bunge*, Stellaria dichotoma* L. var. *lanceolata* Bge.*, Paeonia lactiflora* Pall*, Gastrodia elata* Bl.*, Uncaria rhynchophylla* (Miq.) Jacks*, Acorus gramineus* Soland.*, Ampelopsis japonica* (Thunb.) Makino*,* and *Lycopodium clavatum L*. In clinical, QLZDD has been widely used in treating TS for many years and has shown remarkable outcomes. In our previous study, the results of network pharmacology and molecular docking revealed that otophylloside B, quercetin, yohimbin and otophylloside A may be the material basis of QLZDD, which play an anti-TS role by regulating inflammatory factors, neuroimmunity and neurotransmitters. However, the lack of modern scientific support hinders the application and the promotion of QLZDD. In the present study, we aimed to evaluate the effect and to explain the underlying mechanism of the effect of QLZDD on neurotransmitters and intestinal flora using a TS mouse model.

## Material and Methods

### Main Materials

3,3-Iminodipropionitrile (IDPN, batch no. 317306) was purchased from Sigma-Aldrich Co., Ltd. (United States). Haloperidol was obtained from Shanghai Xinyi Pharmaceutical Co., Ltd. (lot no. H31021234, Shanghai, China) and was used as the positive control drug. ELISA kits were purchased from Shanghai Jining Industrial Co., Ltd. (code no. A05102 for DA; code no. N03931 for GABA; and code no. A06258 for Glu, Shanghai, China). The primary antibodies were GABA-AR alpha 4 (code no. NBP2-59326), dopamine D1R/DRD1 (SG2-D1a, code no. NB110-6007), D2DR (B10, code no. sc-5303), and DAT (code no. 22524-1-AP) antibodies.

### Animals

A total of 90 male Kunming (KM) mice (SPF, 18–22 g) that were used in this study were purchased from the Animal Experimental Center of Kunming Medical University (license no. SCXK (Yunnan) K2015-002). The animals were housed in the Laboratory Animal Center of Yunnan University of Chinese Medicine under standard laboratory condition (22 ± 2°C, 50 ± 10% relative humidity, 12-h-light/12-h-dark cycle) and were given ad libitum access to water. Before experiments, all mice were accustomed to adaptive feeding for one week. The experiments were conducted under the full authorization from the Ethics Committee of Yunnan University of Chinese Medicine (ethical code no. R-06202044).

### Preparation of QLZDD

The formula of QLZDD consists of 9 herbs (Table 1) that were purchased from the Department of Pharmacy of the First Clinical Medical College of Yunnan University of Traditional Medicine. Firstly, the herbs were extracted with 10 times the amount of water for 2 h, and the herb extracts were filtered for the preparation of QLZDD granules. After identification, the herbs were decocted twice with water (2 h each time), then centrifuged, filtered, and concentrated under low temperature and concentration. By adding the appropriate amount of spray-dried lactose, we made 60 g of QLZDD granules containing 1.75 g of raw drug per gram of the granule. Then, the components and the active components of QLZDD were detected by LC-MS.

**TABLE 1:** The composition and the relevant information on the components of QLZDD.

Herbs	Voucher specimen number	Part(s) used	Medicinal amount (g)
*Cynanchum otophyllum* C.K. Schneid	130301	roots	10
*Gentiana scabra* Bunge	1911012	roots and root stems	5
*Stellaria dichotoma* L. var. *lanceolata* Bge	190901	roots	10
*Paeonia lactiflora* Pall	20200202	roots	10
*Gastrodia elata* Bl	1911007	rhizomes	15
*Uncaria rhynchophylla* (Miq.) Jacks	190701501	stem branches with hooks	15
*Acorus gramineus* Soland	20190901	rhizomes	15
*Ampelopsis japonica* (Thunb.) Makino	190501	root tubers	10
*Lycopodium clavatum* L	20191201	whole grass with roots	15

### The Identification of the Compounds in QLZDD by UHPLC-MS/MS

Vanquish™ ultra—high-performance liquid chromatography (UHPLC) system (Thermo Scientific, United States) was used to analyze the compounds in QLZDD. The chromatographic column was Hyperil Gold column (C_18_, 2.1 mm × 100 mm, 1.9 μm), mobile phase A was 0.1% aqueous formic acid solution, and mobile phase B was methanol. The gradient elution condition was shown in Table 2. The column temperature was 40°C, the flow rate was 0.2 ml/min, and the total time was 17 min. The UHPLC system was coupled with a benchtop Q Exactive hybrid quadrupole-Orbitrap mass spectrometer (Thermo Scientific). The positive and the negative ion modes of electrospray ionization source (ESI) were used to collect the mass spectrometric information of QLZDD.

**TABLE 2:** The gradient elution condition.

Time/min	A%	B%
0.0	98	2
1.5	98	2
12.0	0	100
14.0	0	100
14.1	98	2
17.0	98	2

### Experimental Design and Treatment

The mice were randomly divided into six groups including control, model, haloperidol (14 mg/kg), QLZDD low-, middle-, and high-dose groups (6.83, 13.65, and 27.3 g/kg, respectively), and each group had 15 mice. Mice in the control group were intraperitoneally injected with 0.9% saline (3 ml/kg, i.p.); and the others were injected with IDPN (350 mg/kg, i.p.) once a day for 7 consecutive days. After the successful modeling, treatment groups were administered respectively once a day with QLZDD or haloperidol for 4 consecutive weeks, and the control and model group were administered with 0.9% saline (10 ml/kg). The administered dose of QLZDD was calculated by referring to the conventional clinical dosage of children. The time flow chart of the experiment was shown in Figure 2A.

### Behavioral Tests

The stereotyped behavior of the mice was recorded according to the scoring criteria reported previously (Santangelo et al., 2018). Through a transparent box (35 cm length × 25 cm width × 25 cm height), spontaneous motor behavior and the number of Head-Body Twitch (the HBT number) (Chen et al., 2019) were used to evaluate the tic-like behavior of the mice. After the injection of IDPN, a behavioral video of the mice was recorded once a week. The video recording was analyzed by three observers who underwent training and were familiar with the behavioral score, without knowing the grouping of the mice. Each mouse was observed for 30 min, and the HBT number was recorded every 6 min for a total of 5 min.

### Sample Collection and Preparation

Metabolic cages were used to collect fecal samples to assess the effect of QLZDD on gut microbiota. The mice were anesthetized with 1% sodium pentobarbital (50 mg/kg). Blood samples were collected from the abdominal aorta and centrifuged at 1,000×g for 10 min. Then, the superstratum serum was separated and gathered. Finally, the mice were sacrificed, the brains were taken, and the striatum was quickly separated on ice. All specimens were immediately stored at –80 °C until they were used for analysis.

### The Determination of the Content of Neurotransmitters (DA, GABA, and Glu)

The serum and the striatum of the mice were collected, and the level of neurotransmitters (DA, GABA, Glu) in the serum and the striatum were tested by enzyme-linked immunosorbent assay (ELISA). The specific ELISA kit (Shanghai Jining Industrial Co., Ltd., Shanghai, China) was used according to the manufacturer’s instructions.

### Immunohistochemistry

The brain tissue was removed and perfused with 4% formaldehyde and phosphate buffered saline (PBS) (Sigma-Aldrich Corporation). Up to 4% formaldehyde (EM grade) was fixed, dehydrated, and embedded in paraffin. The embedded tissue was cut by a slicer into the pieces with the thickness of 4 μm for immunohistochemical examination. The sections were dewaxed with water and repaired with antigen to block endogenous peroxidase and serum. The sections were sealed with the primary antibody (1:100 D1R, 1:200 D2R, 1:100 GABAR, or 1:200 DAT). Then, the secondary antibody was added. The DAB coloration, the re-staining of the nucleus, and the dehydration of sealing film. The change in the protein expression of DAT, D1R, D2R, and GABAR in the brain striatum was observed, and the morphological images were taken by a slide scanning image analysis system (TEKSQRAY).

### 16s RNA Sequencing and Analysis

After 4 weeks of the drug treatment, the fresh feces of the mice of each group were collected. MO BIO PowerSoil^®^ DNA Isolation Kit was used to extract the DNA from the fecal samples. The amplification primers of the V3 and the V4 regions of the 16s rRNA were (5′-ACT​CCT​ACG​GGA​GGC​AGC​A-3′) and (5′-GGACTACHVGGGTWTCTAAT-3′), respectively. On the basis of Illumina HiSeq sequencing platform, we used the method of double terminal sequencing (paired-end). All the sequences were divided into OTUs by Uparse (version 7.1, http://drive5.com/uparse/). The bioinformatics of the OTUs was analyzed at 97% similarity level, and the representative sequence of the OTUs was compared with the microbial reference database Silva (http://www.ARB-Silva).

On the basis of the clustering results of the OUTs, we conducted beta diversity analysis by using QIIME2 software. Alpha diversity were assessed by the Chao1 and Shannon diversity index. Beta diversity was calculated by the weighted and the unweighted uniform distance, and principal coordinate analysis was carried out. On the basis of the taxonomic information, we carried out species structure analysis and species difference analysis and detected the characteristic of the significant abundance difference to find the groups with the significant abundance difference. By selecting the OTUs or the corresponding sequence of the taxonomic information at a certain level, we constructed an evolutionary tree on the basis of maximum likelihood estimation (MLE) and neighbor joining (NJ) and presented the phylogenetic relationship of the species in a circular graph. The correlation between the species and environmental factors was analyzed by Spearman’s correlation and the significance of the correlation was tested. Selected environmental factors were analyzed by canonical correspondence analysis (CCA) and redundancy analysis (RDA). By calculating the correlation between species, we constructed a species correlation network. The knowledge of graph theory was used to analyze the species correlation network to obtain the relevant intragroup and intergroup information about the species and the samples.

### Statistical Analysis

The data were processed and analyzed by using SPSS 25.0 software, and the results were expressed as mean ± standard deviation ( $\bar{x}$ ± *s*). Repeated measurements were analyzed by repeated measures ANOVA. The remaining data were analyzed by one-way ANOVA. The least significant difference method was used for the *post-hoc* analysis. When the data were not normally distributed, nonparametric tests were used for the item-by-item statistical analysis. For the analysis of gut microbiota, the correlation between factors was calculated by Spearman’s rank sum correlation test, and RDA was performed to analyze the correlation between environmental factors and the microbial community. The alpha level was set to *p* = 0.05, and all *p*-values were generated by two-sided tests.

## Results

### Chemical Analysis of QLZDD

**FIGURE 1:**
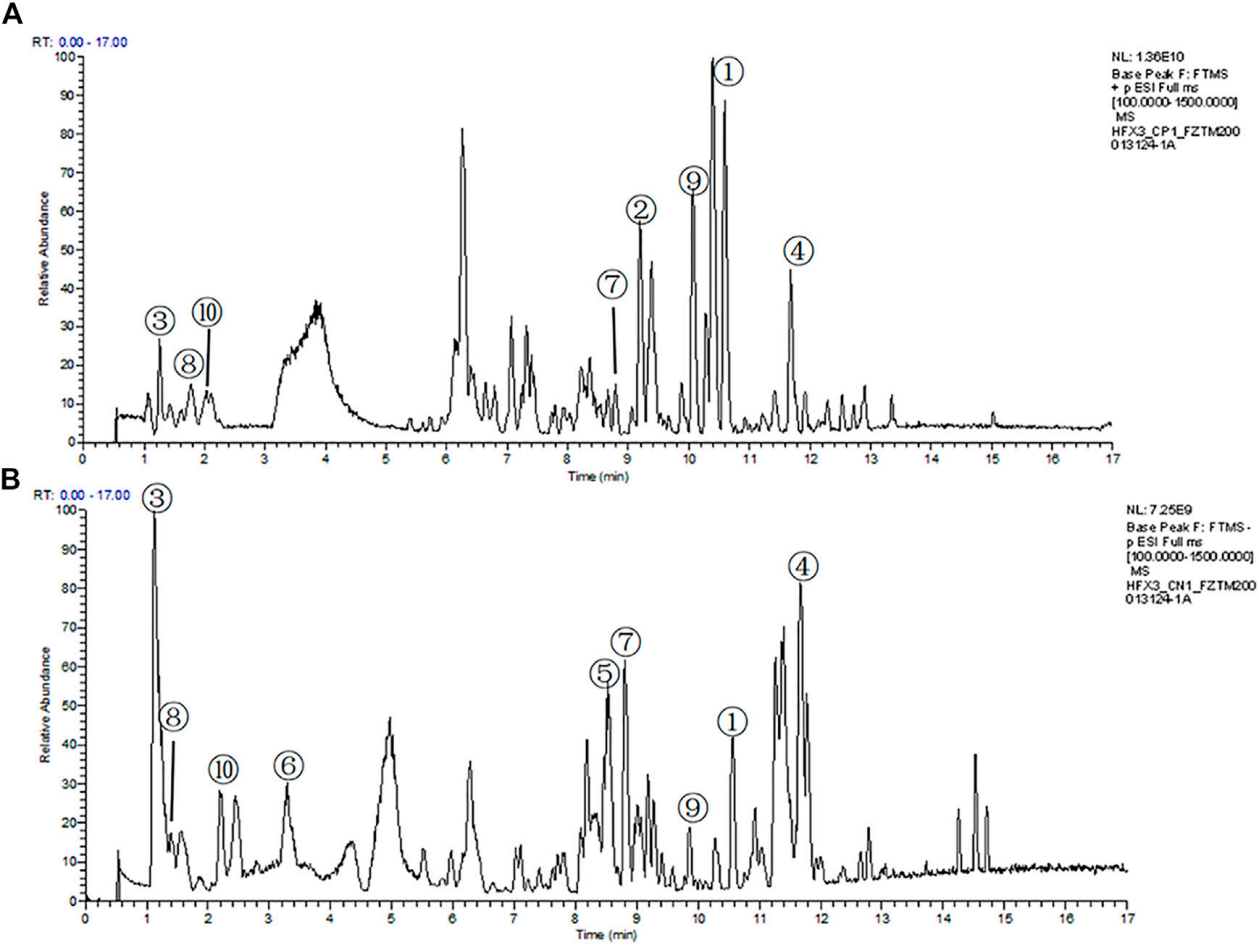
The total ion chromatograms (TIC) of QLZDD that were obtained in **(A)** the positive (ESI^±^) ionization mode and **(B)** the negative (ESI^−^) ionization mode.

The compounds in QLZDD were identified by the UHPLC-MS method, and a total of 291 compounds were characterized in QLZDD by the positive ESI^±^ mode (Figure 1A) or the negative ESI^−^ mode (Figure 1B). The main 10 substances of QLZDD are shown in Table 3. Structural isomers were identified and differentiated on the basis of polarity.

**TABLE 3:** Top 10 substances in QLZDD by a LC-MS method.

Name	Composite score	Formula	Intensity
Hirsutine	0.83	C_22_H_28_N_2_O_3_	54203617924
Isorhynchophylline	0.76	C_22_H_28_N_2_O_4_	37199017647
Citric acid	0.66	C_6_H_8_O_7_	35026590908
3-Hydroxy-5-isopropylidene-3,8-dimethyl-2,3,3a,4,5,8a-hexahydro-6(1H)-azulenone	0.78	C_15_H_22_O_2_	28994570036
Albiflorin	0.66	C_23_H_28_O_11_	27804308371
Parishin E	0.86	C_19_H_24_O_13_	25090194693
Hesperetin	0.62	C_16_H_14_O_6_	23570770912
Quinic acid	0.99	C_7_H_12_O_6_	20648810346
methyl (1S,5R,9S,13R)-5,9-dimethyl-14-methylidenetetracyclohexadecane-5-carboxylate	0.75	C_21_H_32_O_2_	18518566098
Pyroglutamic acid	0.94	C_5_H_7_NO_3_	16972686305

### Effect of QLZDD on Tic-like Behaviors

**FIGURE 2:**
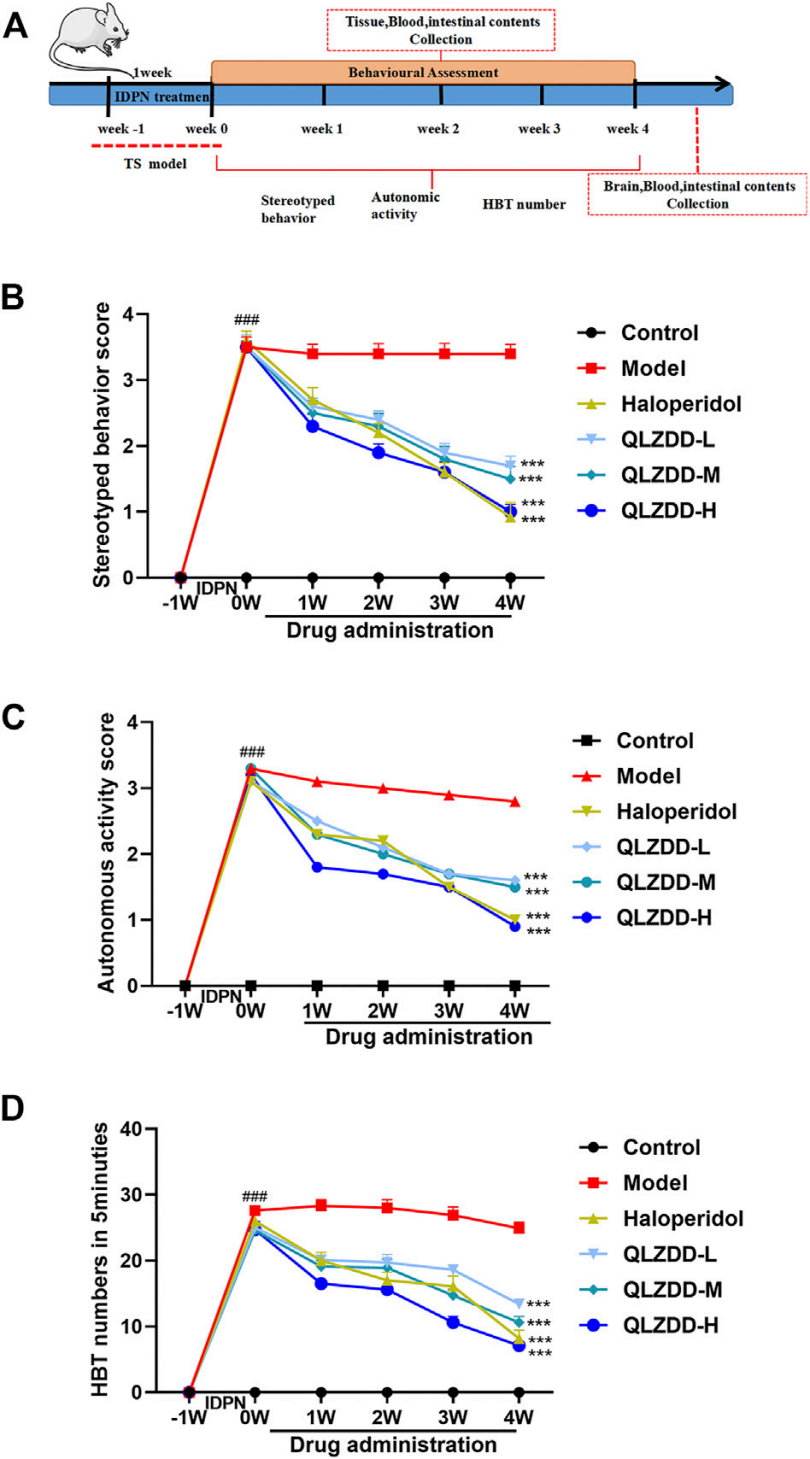
The effect of QLZDD on the tic-like behavior. **(A)** Time flow chart. **(B)** The stereotyped behavior score of the QLZDD groups and the stereotyped behavior score of the haloperidol group were significantly lower than that of the model group. **(C)** The autonomic activity behavior score of the QLZDD groups and the autonomic activity behavior score of the haloperidol group were significantly lower than that of the model group. **(D)** The HBT number of the QLZDD groups and the HBT number of the haloperidol group were significantly lower than that of the model group. All data were expressed as mean ± SD (*n* = 15 mice). The data of the QLZDD groups and the haloperidol group were analyzed by repeated measures ANOVA and were compared to the data of the control group.

During the experiment, the stereotyped behavior score (Figure 2B, F = 354.121, *p* = 5.716 × 10^–40^, η_p_^2^ = 0.970), the autonomic activity behavior score (Figure 2C, F = 177.374, *p* = 3.199 × 10^–32^, η_p_^2^ = 0.943), and the HBT number (Figure 2D, F = 184.418, *p* = 1.190 × 10^–32^, η_p_^2^ = 0.945) changed significantly over time. After 1 week of the IDPN injection, the stereotyped behavior score, the autonomic activity behavior score, and the HBT number of the IDPN-injected group were significantly higher (*p*-values < 0.05) than those of the control group.

After 2 weeks of the drug administration, in comparison with the stereotyped behavior score, the autonomic activity behavior score, and the HBT number of the model group, the stereotyped behavior score (*p* = 2.019 × 10^–4^ for the low-QLZDD-dose group, *p* = 0.012 for the middle-QLZDD-dose group, *p* = 0.035 for the high-QLZDD-dose group, and *p* = 0.006 for the haloperidol group), the autonomic activity behavior score (*p* = 0.070 for the low-QLZDD-dose group, *p* = 0.061 for the middle-QLZDD-dose group, *p* = 0.035 for the high-QLZDD-dose group, and *p* = 0.049 for the haloperidol group), and the HBT number (*p* = 0.013 for the low-QLZDD-dose group, *p* = 0.006 for the middle-QLZDD-dose group, *p* = 7.253 × 10^–5^ for the high-QLZDD-dose group, and *p* = 0.001 for the haloperidol group) of the QLZDD groups and the haloperidol group were significantly lower.

After 4 weeks of the drug administration, in comparison with the stereotyped behavior score, the autonomic activity behavior score, and the HBT number of the model group, the stereotyped behavior score (*p* = 2.137 × 10^–4^ for the low-QLZDD-dose group, *p* = 6.181 × 10^–5^ for the middle-QLZDD-dose group, *p* = 8.120 × 10^–6^ for the high-QLZDD-dose group, and *p* = 1.172 × 10^–4^ for the haloperidol group), the autonomic activity behavior score (*p* = 7.364 × 10^–4^ for the low-QLZDD-dose group, *p* = 6.412 × 10^–5^ for the middle-QLZDD-dose group, *p* = 9.189 × 10^–5^ for the high-QLZDD-dose group, and *p* = 1.384 × 10^–4^ for the haloperidol group), and the HBT number (*p* = 1.868 × 10^–6^ for the low-QLZDD-dose group, *p* = 1.980 × 10^–7^ for the middle-QLZDD-dose group, *p* = 2.119 × 10^–8^ for the high-QLZDD-dose group, and *p* = 2.599 × 10^–6^ for the haloperidol group) of the QLZDD groups and the haloperidol group were significantly lower.

### Effects of QLZDD on DA, Glu and GABA

**FIGURE 3:**
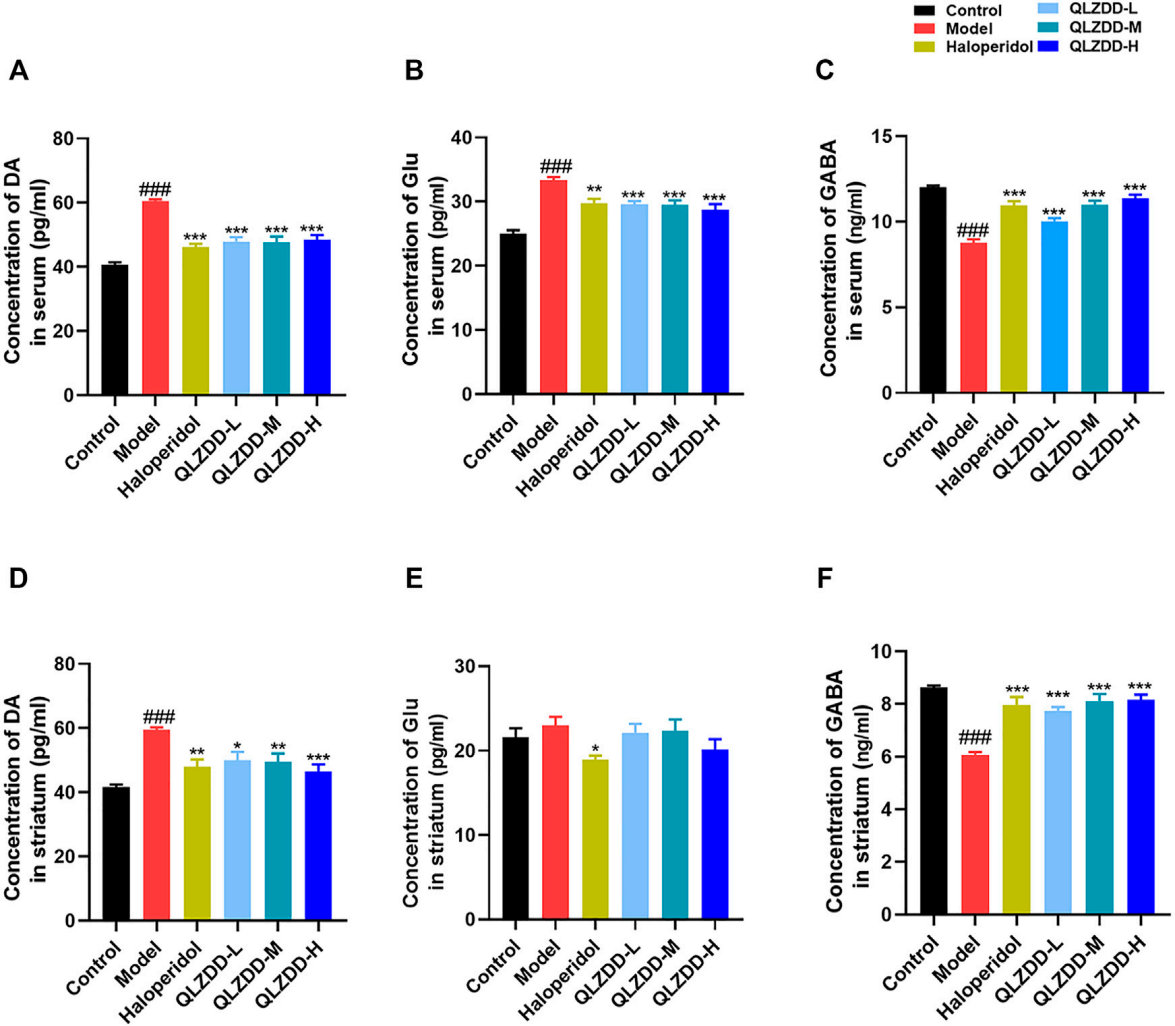
The effect of QLZDD on DA, Glu, and GABA. **(A)** The level of the DA expression in serum, **(B)** the level of the Glu expression in serum, **(C)** the level of the GABA expression in serum, **(D)** the level of the DA expression in the striatum, **(E)** the level of the Glu expression in the striatum, and **(F)** the level of the GABA expression in the striatum. All data were expressed as mean ± SD (*n* = 8 mice). One-way ANOVA was used to compare the data of the QLZDD groups to the data of the control group. ^###^ *p* < 0.001 in comparison with the model group. *p < 0.05, ***p < 0.01, and ***p < 0.001.

IDPN significantly increased DA (Figure 3A, *p* = 1.004 × 10^–14^), Glu (Figure 3B, *p* = 2.291 × 10^–11^), while significantly decreased GABA (Figure 3C, *p* = 8.417 × 10^–15^). After 4 weeks of the drug administration, in comparison with the DA and the Glu of the model group, the DA (*p* = 3.937 × 10^–9^ for the low-QLZDD-dose group, *p* = 2.270 × 10^–9^ for the middle-QLZDD-dose group, *p* = 1.418 × 10^–8^ for the high-QLZDD-dose group, and *p* = 1.214 × 10^–10^ for the haloperidol group) and the Glu (*p* = 1.914 × 10^–4^ for the low-QLZDD-dose group, *p* = 1.620 × 10^–4^ for the middle-QLZDD-dose group, *p* = 1.129 × 10^–5^ for the high-QLZDD-dose group, and *p* = 4.072 × 10^–4^ for the haloperidol group) of drug-treated groups were significantly lower. On the other hand, the GABA of drug-treated groups was higher than that of the model group (*p* = 4.699 × 10^–5^ for the low-QLZDD-dose group, *p* = 4.945 × 10^–10^ for the middle-QLZDD-dose group, *p* = 7.059 × 10^–12^ for the high-QLZDD-dose group, and *p* = 8.102 × 10^–10^ for the haloperidol group).

IDPN significantly increased DA (Figure 3D, *p* = 4.700 × 10^–7^), Glu (Figure 3E, *p* = 0.809), while significantly decreased GABA (Figure 3F, *p* = 1.236 × 10^–10^). After 4 weeks of the drug administration, in comparison with the DA, the Glu, and the GABA of the model group, the DA (*p* = 2.385 × 10^–3^ for the low-QLZDD-dose group, *p* = 1.487 × 10^–3^ for the middle-QLZDD-dose group, *p* = 7.479 × 10^–5^ for the high-QLZDD-dose group, and *p* = 3.713 × 10^–4^ for the haloperidol group) of drug-treated groups were significantly lower, the Glu of the QLZDD-treated groups was not significantly lower (*p* = 0.966 for the low-QLZDD-dose group, *p* = 0.989 for the middle-QLZDD-dose group, *p* = 0.219 for the high-QLZDD-dose group, and *p* = 0.043 for the haloperidol group), and the GABA of drug-treated groups were significantly higher (*p* = 1.132 × 10^–6^ for the low-QLZDD-dose group, *p* = 2.423 × 10^–8^ for the middle-QLZDD-dose group, *p* = 1.391 × 10^–8^ for the high-QLZDD-dose group, and *p* = 1.085 × 10^–7^ for the haloperidol group).

### Effects on the mRNA Expression of D1R, D2R, DAT, and GABAR

**FIGURE 4:**
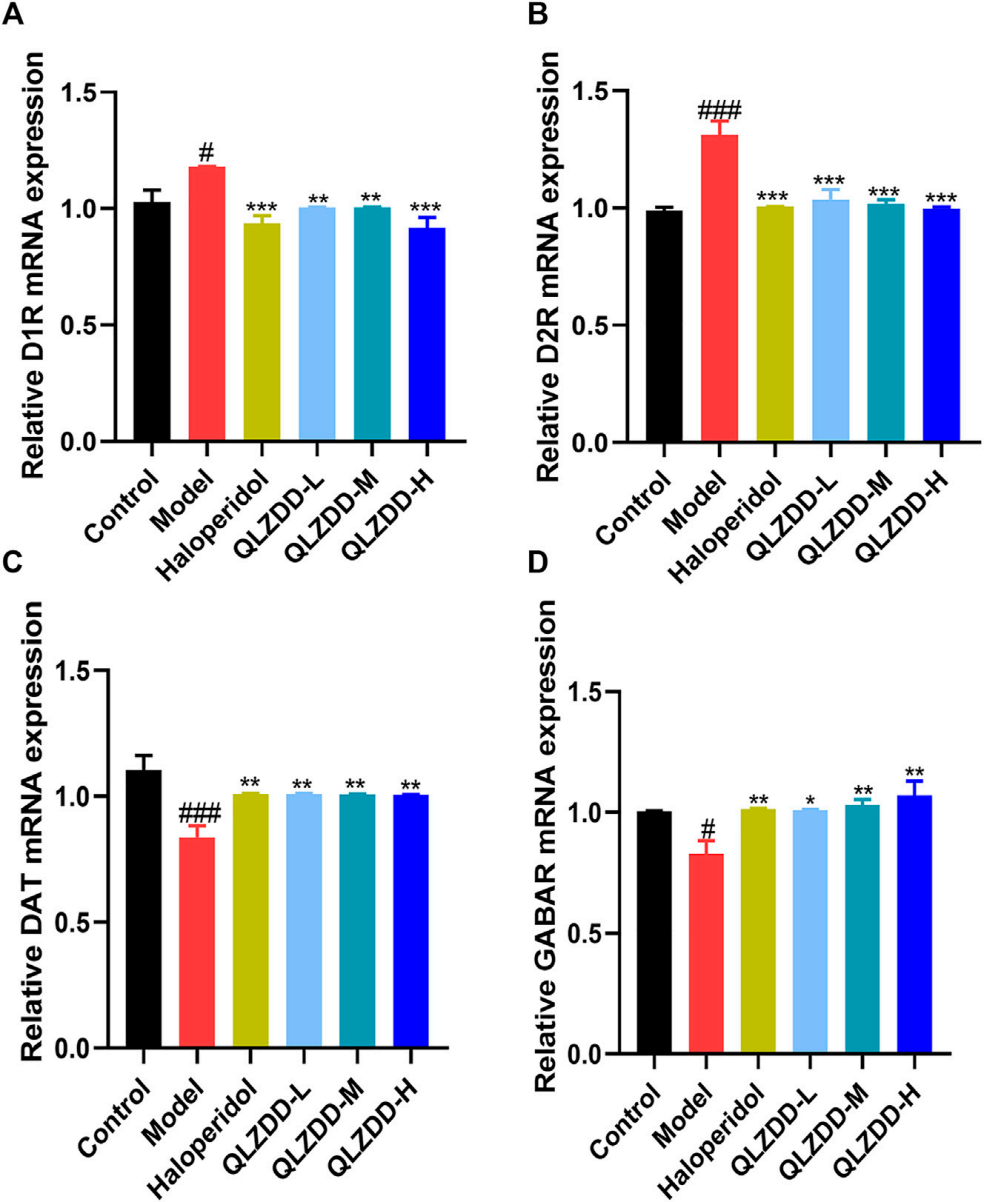
The effect of QLZDD on the mRNA expression of D1R, D2R, DAT, and GABAR. **(A)** The level of the D1R mRNA expression in serum, **(B)** the level of the D2R mRNA expression in serum, **(C)** the level of the DAT mRNA expression in serum, and **(D)** the level of the GABAR mRNA expression in serum. All data were expressed as mean ± SD (*n* = 3 mice). One-way ANOVA was used to compare the data of the QLZDD groups with the data of the control group. ^###^ *p* < 0.001 in comparison with the model group. *p < 0.05, ***p < 0.01, and ***p < 0.001.

IDPN significantly increased the level of D1R (Figure 4A, *p* = 0.018), D2R (Figure 4B, *p* = 9.273 × 10^–6^), while significantly decreased the level of DAT (Figure 4C, *p* = 0.002), and GABAR (Figure 4D, *p* = 0.013) in the striatum.

After 4 weeks of the drug administration, in comparison with the D1R, the D2R, the DAT, and the GABAR of the model group, the D1R (*p* = 0.001 for the low-QLZDD-dose group, *p* = 0.007 for the middle-QLZDD-dose group, *p* = 0.007 for the high-QLZDD-dose group, and *p* = 0.003 for the haloperidol group) and the D2R (*p* = 4.233 × 10^–5^ for the low-QLZDD-dose group, *p* = 2.382 × 10^–5^ for the middle-QLZDD-dose group, *p* = 1.215 × 10^–5^ for the high-QLZDD-dose group, and *p* = 1.577 × 10^−5^for the haloperidol group) of drug-treated groups were significantly lower, while the DAT (*p* = 0.007 for the low-QLZDD-dose group, *p* = 0.008 for the middle-QLZDD-dose group, *p* = 0.008 for the high-QLZDD-dose group, and *p* = 0.007 for the haloperidol group) and the GABAR (*p* = 0.012 for the low-QLZDD-dose group, *p* = 0.005 for the middle-QLZDD-dose group, *p* = 0.001 for the high-QLZDD-dose group, and *p* = 0.009 for the haloperidol group) of drug-treated groups were significantly higher.

### Effects on the Protein Expression of D1R, D2R, DAT, and GABAR

**FIGURE 5:**
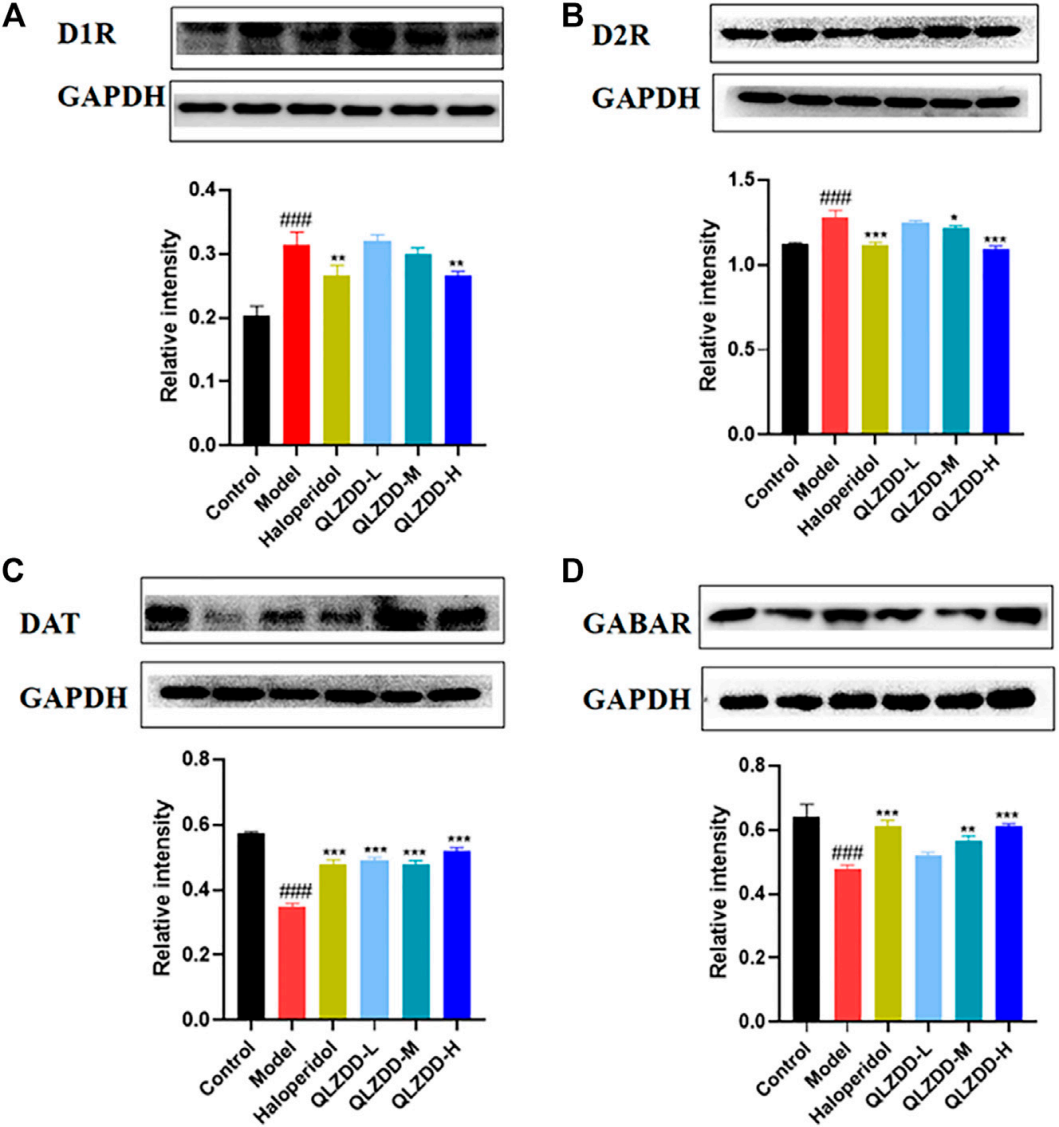
The effect of QLZDD on the protein expression of D1R, D2R, DAT, and GABAR. **(A)** The level of the D1R protein expression in serum, **(B)** the level of the D2R protein expression in serum, **(C)** the level of the DAT protein expression in serum, and **(D)** the level of the GABAR protein expression in serum. All data were expressed as mean ± SD (*n* = 3 mice). One-way ANOVA was used to compare the data of the QLZDD groups with the data of the control group. ^###^ *p* < 0.001 in comparison with the model group. *p < 0.05, ***p < 0.01, and ***p < 0.001.

IDPN significantly increased the level of D1R (Figure 5A, *p* = 4.44 × 10^–7^), D2R (Figure 5B, *p* = *p*=1.91 × 10^–6^), while significantly decreased the level of DAT (Figure 5C, *p* = 7.33 × 10^–12^), and GABAR (Figure 5D, *p* = 5.96 × 10^–7^) in the striatum.

After 4 weeks of the drug administration, in comparison with the D1R, the D2R, the DAT, and the GABAR of the model group, the D1R (*p* = 0.563 for the low-QLZDD-dose group, *p* = *p*=0.258 for the middle-QLZDD-dose group, *p* = 0.001 for the high-QLZDD-dose group, and *p* = 0.001 for the haloperidol group) and the D2R (*p* = *p*=0.16 for the low-QLZDD-dose group, *p* = *p*=0.01 for the middle-QLZDD-dose group, *p* = *p*=2.34 × 10^–7^ for the high-QLZDD-dose group, and *p* = 1.22 × 10^–6^ for the haloperidol group) of drug-treated groups were significantly lower, while the DAT (*p* = 1.55 × 10^−9^for the low-QLZDD-dose group, *p* = 3.55 × 10^–9^ for the middle-QLZDD-dose group, *p* = 1.71 × 10^–10^ for the high-QLZDD-dose group, and *p* = 4.74 × 10^–9^ for the haloperidol group) and the GABAR (*p* = 0.035 for the low-QLZDD-dose group, *p* = 2.35 × 10^–4^ for the middle-QLZDD-dose group, *p* = 5.21 × 10^–6^ for the high-QLZDD-dose group, and *p* = 5.21 × 10^–6^ for the haloperidol group) of drug-treated groups were significantly higher.

### Effect of QLZDD on D1R, D2R, DAT and GABAR

**FIGURE 6:**
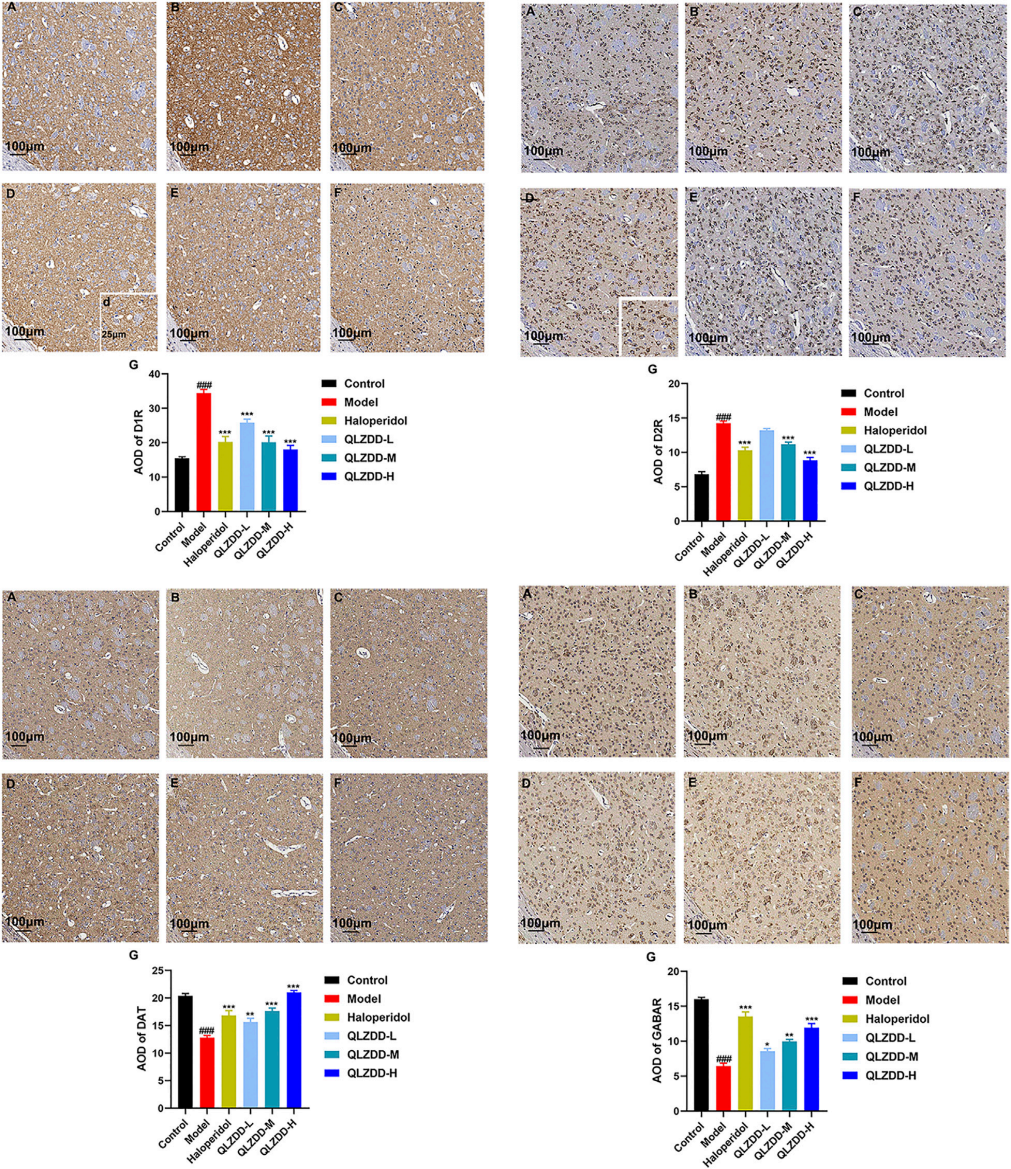
The immunohistochemistry of D1R, D2R, DAT, and GABAR. **(A)** The control group, **(B)** the model group, **(C)** the haloperidol group, **(D)** the low-QLZDD-dose group, **(E)** the middle-QLZDD-dose group, **(F)** the high-QLZDD-dose group, and **(G)** the histogram of the immunohistochemistry. All data were expressed as mean ± SD (*n* = 5 mice). One-way ANOVA was used to compare the data of the model group, the haloperidol group, and the QLZDD groups with the data of the control group. ^###^: *p* < 0.001 in comparison with the model group. ***: *p* < 0.001. Photos were taken with ×40 and ×10 magnification.

IDPN significantly increased the expression of D1R (*p* = 1.229 × 10^–10^ for the model group versus the control group) and D2R (*p* = 1.924 × 10^–13^ for the model group versus the control group) in the striatum (Figures 6A,B) and significantly decreased the expression of DAT (*p* = 1.927 × 10^–5^ for the model group versus the control group) and GABAR (*p* = 9.369 × 10^–14^ for the model group versus the control group) in the striatum (Figures 6C–F). After 4 weeks of the drug administration, in comparison with the expression of D1R in the striatum, the expression of D2R in the striatum, the expression of DAT in the striatum, and the expression of GABAR in the striatum of the model group, the expression of D1R in the striatum of the QLZDD groups (*p* = 6.752 × 10^–5^ for the low-QLZDD-dose group, *p* = 2.898 × 10^–9^ for the middle-QLZDD-dose group, and *p* = 1.563 × 10^–8^ for the high-QLZDD-dose group) and the haloperidol group (*p* = 2.914 × 10^–8^) were significantly lower, the expression of D2R in the striatum of the QLZDD-treated groups (*p* = 0.005 for the low-QLZDD-dose group, *p* = 2.882 × 10^–6^ for the middle-QLZDD-dose group, and *p* = 1.385 × 10^–10^ for the high-QLZDD-dose group) and the haloperidol group (*p* = 5.613 × 10^–8^) were significantly lower, the expression of DAT in the striatum of the QLZDD groups (*p* = 0.012 for the low-QLZDD-dose group, *p* = 0.068 for the middle-QLZDD-dose group, and *p* = 0.991 for the high-QLZDD-dose group) and the haloperidol group (*p* = 0.148) were significantly higher, and the expression of GABAR in the striatum of the QLZDD groups (*p* = 0.002 for the low-QLZDD-dose group, *p* = 9.250 × 10^–6^ for the middle-QLZDD-dose group, and *p* = 7.159 × 10^–9^ for the high-QLZDD-dose group) and the haloperidol group (*p* = 5.020 × 10^–11^) were significantly higher.

### Effects of QLZDD on the Composition of Gut Microbiota

The Alpha diversity can reflect the species abundance (Chao1 index) and the species diversity (Shannon index) within the samples. The Chao1 index showed a significant increase in the QLZDD group (*p* = 0.034), suggesting an increase in the total number of OUTs in the high-dose QLZDD group. The Shannon index results showed a significant decrease in the high-dose QLZDD group compared with the model group (*p* = 0.013).

**FIGURE 7:**
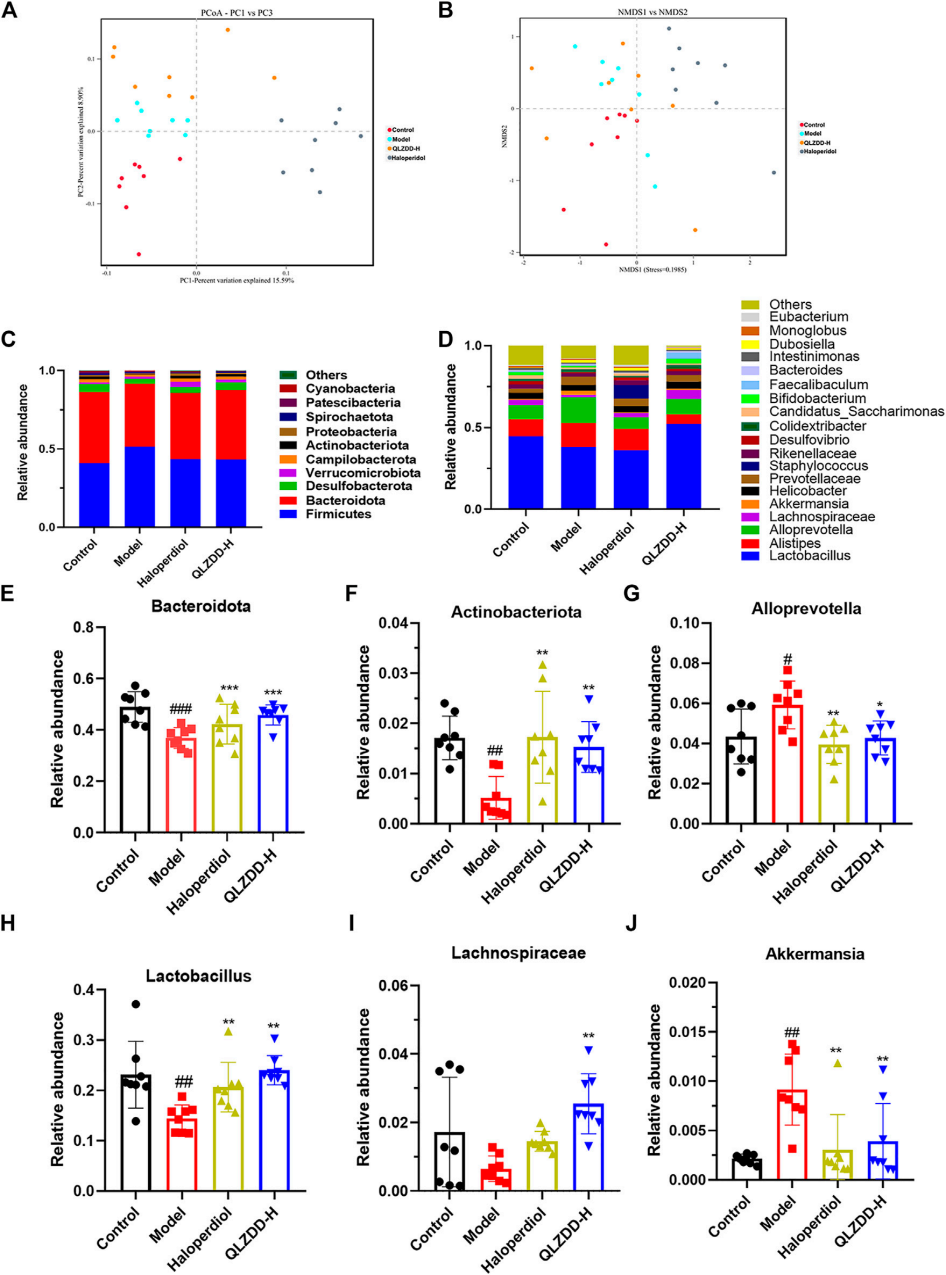
The effect of QLZDD on the composition of gut microbiota. **(A)** The PCoA of samples at the OTU level, **(B)** the NMDS ordination of samples at the OTU level, **(C)** the relative abundance of the 10 main phyla, **(D)** the relative abundance of the 20 main genera, **(E, F)** the changes in different microflora at the phylum level, and (**G–J**) the changes in different microflora at the genus level. All data were expressed as mean ± SD (*n* = 8 mice). One-way ANOVA was used to compare the data of the QLZDD groups with the data of the control group. ^##^ *p* < 0.001 in comparison with the model group. *p < 0.001.

*Beta diversity analysis* included principal coordinate analysis (PCoA) and non-metric multidimensional scaling (NMDS) analysis. PCoA revealed the relative difference in the sequence similarity between strains. NMDS, which is based on evolutionary relationships or quantitative distance matrices, was used to compare the differences between samples or groups. Except for individual samples, there are significant differences among the four groups (Figures 7A,B).

### Multi-Sample Species Classification

The microbial diversity analysis indicated that the intestine microbiota included the following 10 major phylum (Figure 7C): *Firmicutes*, *Bacteroidota*, *Desulfobacterota*, *Verrucomicrobiota*, *Campilobacterota*, *Actinobacteriota*, *Proteobacteria*, *Spirochaetota*, *Patescibacteria*, *Cyanobacteria*. *Bacteroidetes*, *Firmicutes*, *Verrucomicrobia*, *Proteobacteria* were the dominant bacteria at the phylum level (>90%).

IDPN increases the abundance of Firmicutes (*p =* 0.723) and significantly decreases the abundance of Bacteroidetes (*p* = 1.990 × 10^–4^) (Figure 7E) and Actinobacteria (*p* = 0.001) (Figure 7F). After 4 weeks of the drug administration, in comparison with the abundance of Firmicutes, Bacteroidetes, and Actinobacteria of the model group, the abundance of Firmicutes of drug-treated groups is lower (*p* = 0.847 for the high-QLZDD-dose group and *p* = 0.841 for the haloperidol group), and the abundance of Bacteroidetes (*p* = 0.004 for the high-QLZDD-dose group and *p* = 0.067 for the haloperidol group) and Actinobacteria (*p* = 0.006 for the high-QLZDD-dose group and *p* = 0.001 for the haloperidol group) of drug-treated groups is higher (Figure 7C).

At the genus level, 30 dominant genera are diagnosed in the four groups (Figure 7D), including *Lactobacillus*, *Alloprevotella*, *Akkermansia*, *Alistipes*, *Blautia*, *Odoribacter*, *Turicibacter*, *Bacteroides*, *Roseburia*, and *Mucispirillum*. IDPN increases the abundance of *Alloprevotella* (*p* = 0.022, Figure 7G) and *Akkermansia* (*p* = 0.004, Figure 7J) and decreases the abundance of *Bacteroides* (*p* = 0.001, Figure 7E), *Lactobacillus* (*p* = 0.002, Figure 7H), and *Lachnospiraceae* (*p* = 0.007, Figure 7I).

After 4 weeks of the drug administration, in comparison with the abundance of *Lactobacillus*, *Bacteroides*, and *Lachnospiraceae* of the model group, the abundance of *Lactobacillus* (*p* = 0.001 for the high-QLZDD-dose group and *p* = 0.003 for the haloperidol group), *Bacteroides* (*p* = 0.002 for the high-QLZDD-dose group and *p* = 0.055 for the haloperidol group), and *Lachnospiraceae* (*p* = 0.001 for the high-QLZDD-dose group and *p* = 0.230 for the haloperidol group) of the drug-treated groups is higher. The abundance of *Akkermansia* (*p* = 0.007 for the high-QLZDD-dose group and *p* = 0.002 for the haloperidol group) and *Alloprevotella* (*p* = 0.016 for the high-QLZDD-dose group and *p* = 0.004 for the haloperidol group) of the drug-treated groups is lower than that of the model group (Figure 7F).

### LEfSe Analysis Revealed the Specific Gut Microbiota

**FIGURE 8:**
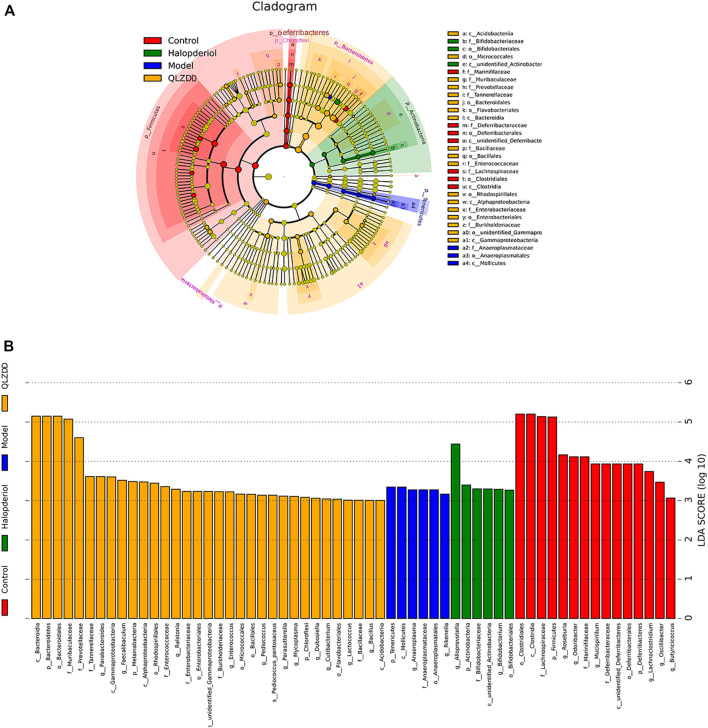
LEfSe analysis revealed the specific gut microbiota. **(A)** LEfSe analysis of evolutionary branching graphs. The circle of evolutionary branching maps from internal to external radiation represents the classification level from the phyla to the species. A significant value of less than 0.05 was used as a threshold for the LEfSe analysis. **(B)**The LDA score obtained from LEfSe analysis of gut microbiota in different groups. A LDA effect size of more than 3 was used as a threshold for the LEfSe analysis.

LefSe analysis was applied to estimate the effect of species richness in each group on diversity, and to identify communities or species that were significantly different in sample allocation. There were 60 LDA scores> 3 in the control group, model group, haloperidol group, and QLZDD high-dose group. It found 6 bacterias in the model group, *Tenericutes*, *Mollicutes*, *Anaeroplasmatales*, and *Anaeroplasmataceae*. A total of 33 consortia of *Bacteroidetes*, *Bacteroidia*, *Bacteroidales*, *Muribaculaceae*, *Prevotellaceae*, and *Tannerellaceae* were identified in the QLZDD high-dose group. *Alloprevotella*, *Actinobacteria*, *Bifidobacteriaceae*, *Bifidobacterium*, and *Bifidobacteriales* were identified in the haloperidol group (Figures 8A,B).

### Correlation Analysis Between Gut Microbiota and Environmental Factors (DA, Glu, GABA)

**FIGURE 9:**
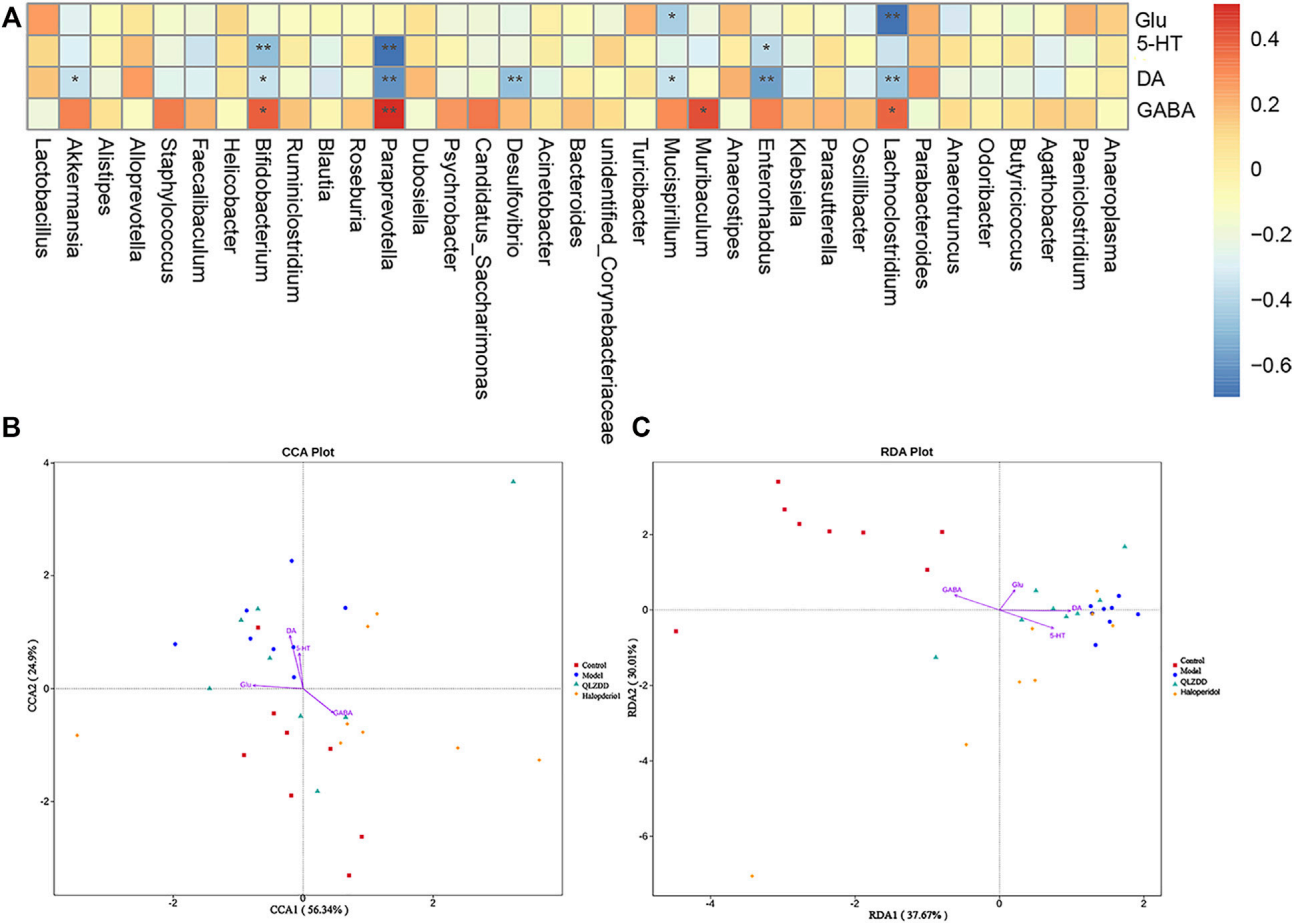
Correlation analysis between gut microbiota and environmental factors (DA, Glu, GABA). **(A)** The correlation analysis map of the striatum biochemical index and the intestinal flora level of the mice. Each row of the correlation analysis map represents a neurotransmitter factor. The color-coded temperature is the Spearman’s correlation coefficient of *n* = 8 mice. *p < 0.05, **p < 0.01, and ***p < 0.001. **(B, C)** The RDA) and the CCA of intracellular bacterial OTUs.

Spearman’s correlation was used to rank the two variables and to perform linear correlation analysis to study the relationship and to obtain the correlation coefficient between the environmental factors and the abundance of microbial species. CCA and RDA were used to reflect the relationship between the microflora and the environmental factors (Figures 9B,C). QLZDD significantly changed the composition of intestinal microflora and improved the level of neurotransmitters in the peripheral and the central nervous system of the TS-induced mice. We used Spearman’s correlation to further analyze the correlation between the composition of intestinal microflora and the level of neurotransmitters (Figure 9A). There was a negative correlation between DA and *Akkermansia* and a negative correlation between DA and *Desulfovibrio* (*p*-values < 0.05). There was a negative correlation between Glu and *Bifidobacterium*, a negative correlation between Glu and *Psychrobacter*, and a negative correlation between Glu and *Enterorhabdus* (*p-*values < 0.05). There was a positive correlation between GABA and *Bifidobacterium*, a positive correlation between GABA and *Psychrobacter*, and a positive correlation between GABA and *Enterorhabdus* (*p-*values < 0.05). There was a positive correlation between Glu and *Mucispirillum* and a positive correlation between DA and *Lachnoclostridium* (*p-*values < 0.05). It can be seen that DA, GABA and Glu neurotransmitters are closely related to gut microbiota (Figures 9A–C).

## Discussion

Our previous clinical study had found that there were differences in the composition of gut microbiota between TS children and healthy children. Our results showed that the beta diversity of bacteria is mainly manifested by the decrease of beneficial bacteria and increased harmful bacteria in the TS group, such as *Bacteroide*, *Lactobacillus*, *Blautia*, and *Roseburia*, and the relatively increased abundance of *Alistipes* and *Akkermansia*. In the current study, an IDPN-induced-TS mouse model was used to evaluate the potential of QLZDD to alleviate the tic-like behavior by regulating the composition of gut microbiota and the level of neurotransmitters. Our TS-induction procedure could mediate the stereotyped behavior and autonomic activity behavior in the mice. Based on previous research, the manipulation *via* injecting IDPN would simulate the neurological syndromes caused by irregular neurotransmission (Long et al., 2017). Therefore, the TS model could serve as a feasible way to explore the potential of QLZDD on the neurological syndromes.

The results showed that QLZDD ameliorated the tic-like behavior and regulated intestinal flora, increased the level of DA and Glu in serum and the striatum and decreased the level of GABA significantly. Furthermore, QLZDD increased the mRNA and protein expression of D1R and D2R, while decreased the mRNA and protein expression of DAT and GABAR in the striatum. The present study showed that QLZDD alleviated TS through modulating the neurotransmitters.

Previous research has indicated the association between intestinal flora and behavior (Barrett et al., 2012). Clinical studies have found that TS patients have the symptoms of intestinal flora disorders such as constipation and dyspepsia, which are related to intestinal flora imbalance. In our previous study, it was found that there were significant differences in the alpha diversity of the intestinal microflora between the TS patient group and the healthy children group. The intestinal microflora diversity of the TS patients increases and manifests by an increase in the number of harmful bacteria (*Alloprevotella, Alistipes,* and *Akkermansia*) and a decrease in the number of beneficial bacteria (*Lactobacillus* and *Bacteroides*). In this study, after the intraperitoneal injection of IDPN, the intestinal flora of the mice changed significantly. In comparison with the abundance of *Alistipes*, *Paraprevotella*, *Bacteroides*, *Alloprevotella*, and *Akkermansia* of the control group, the abundance of *Alistipes*, *Paraprevotella*, and *Bacteroides* of the model group were lower and the abundance of *Alloprevotella* and *Akkermansia* of the model group were higher. The abundance of *Actinobacteria*, *Lactobacillus*, and *Bacteroides* of the haloperidol group and the QLZDD groups was higher than that of the model group, and the abundance of *Alloprevotella* and *Akkermansia* of the haloperidol group and the QLZDD groups was lower than that of the model group. Researchers reported that *Lactobacillus* and *Bifidobacterium* can produce GABA (Dinan et al., 2013), and *Escherichia*, *Bacillus*, and *Saccharomyces* can produce norepinephrine. Other researchers reported that *Bacillus* can produce dopamine and *Lactobacillus* can produce acetylcholine (Bercik et al., 2011). In animal studies, numerous bacterial strains can mediate the behavioral effect by affecting the vagus nerve (Penzol et al., 2019).

Moreover, the regulation of neurotransmitter production is a possible mechanism by which intestinal microbiota affects the brain, which is related to the occurrence of TS. A large number of studies have shown that the gut and brain form the gut-brain axis through two-way neural, hormonal, and immune regulation (Petra et al., 2015). Intestinal microbes affect neurotransmitter-related metabolites and cross the blood-brain barrier and affect the function of the central nervous system (Briguglio et al., 2018). For example, intestinal microbes affect GABA (Thomas et al., 2012; Lyte et al., 2018) and DA, which are important neurotransmitters or neurotransmitter precursors in the brain (Wall et al., 2014). On the basis of our results, we found a negative correlation between DA and *Akkermansia*, a negative correlation between Glu and *Bifidobacterium*, and a positive correlation between GABA and *Bifidobacterium*. It revealed that neurotransmitters DA, GABA, and Glu may be closely related to gut microbiota. The intestinal flora in the body plays a vital role in the effects of traditional Chinese medicine entering the body, and its corresponding effective components to maintain the balance of gut microbiota in the body. Exploring the role and mechanism of traditional Chinese medicine through the gut microflora has become a research hotspot in the treatment of disease, included neurological disorders (Du et al., 2020; Li et al., 2020). Based on above results, we speculated that QLZDD protected the brain function and behavior by regulating the composition of intestinal flora.

As is known to all DA is a neurotransmitter released by the nerve ending of midbrain neurons, and it holds the aspects of neural function (exercise, mood, sleep, etc.) (Zawilska, 2003). DA participates in the activation of the dorsolateral prefrontal cortex, the striatum, and the thalamus and the regulation of the cortical connection to the basal ganglia (Plessen et al., 2007). The use of dopamine receptor antagonists can effectively relieve the clinical symptoms of the patients with TS (Pringsheim et al., 2019a). D1R is a highly sensitive and expressive indicator of direct pathway striatal neurons. It promotes the DA and D1R binding, which can induce the stereotyped behavior (e.g., sniffing, biting, gnawing, and licking) in mice (Fabricius et al., 2017). D2R is widely discovered in medium spiny neurons (MSN) and DA neurons in the basal ganglia and is an essential target for the treatment of the disorders exhibiting aberrant DA functions such as TS and schizophrenia. The increase in the D2R sensitivity and the DA stimulates the excitatory behavior of mice and aggravates TS symptoms. DAT can regulate the level of extracellular dopamine (Gainetdinov et al., 1998). The expression of DAT can directly have an effect on the DA synaptic level. Therefore, DAT performs a key role in the regulation of the DA transmission. After uptaken by DAT, DA is transformed into HVA in neurons and is released into blood, which reduces the DA content in brain tissue or promotes the metabolism of DA, inhibits the activity of the DA receptor, and controls the TS behavior. As reported, DAT will lead to a significant decrease in the striatum of TS patients. And the decrease of DAT will decrease the content of intracellular DA while increase the extracellular DA. DAT knockout mice show excessive DA energy syndromes and the stereotyped behavior (Berridge et al., 2005). The content and the activity of DAT in the striatum and the density and the sensitivity of D2R and D1R are closely related to the pathogenesis of TS (Segura and Strafella, 2013). Our results showed that QLZDD decreased the mRNA expression and the protein expression of D1R and D2R and increased the mRNA expression and the protein expression of DAT in the striatum of the IDPN-induced-TS mice.

GABA and Glu are highly expressed in brain tissue and are regularly associated with nervous system abnormalities (Butler et al., 2018). The deficiency in the GABA inhibitory transmission is related to the pathogenesis and the symptom severity of TS (Tian et al., 2016). Glu is an excitatory neurotransmitter and GABA is an inhibitory neurotransmitter (O'Neill et al., 2019). Glu and GABA participate in the neurophysiological response of neurons to tactile stimuli. The insufficient inhibition of GABA leads to the loss of cerebral cortical function and neuronal excitotoxicity. In clinical settings, the scanning of TS patients by 11C-flumazenil positron emission showed that the GABAR binding potential decreases in TS patients (Lerner et al., 2012). We speculated that QLZDD improved the stereotyped behavior of mice by reducing the mRNA expression and the protein expression of D1R and D2R, reducing the content of DA and Glu, increasing the mRNA expression and the protein expression of DAT and GABAR, and increasing the content of GABA. Our results showed that in comparison with the level of GABA and the level of Glu of the control group, the level of GABA of the QLZDD groups was higher and the level of Glu of the QLZDD groups was lower. In addition, QLZDD increased the mRNA expression and the protein expression of GABAR in the striatum of the IDPN-induced-TS mice. These results indicated that QLZDD improved the flexibility and adaptability, and attenuated the damage in IDPN-induced-TS model.

Collectively, QLZDD could be an effective and promising agent for the treatment of the IDPN-induced TS, which might exert its action by regulating the composition of gut microbiota and the level of neurotransmitters. In addition, there was a limitation of the experiment involving the small striatum in the brain tissue of the mice which was difficult to be located accurately. Our study provides new insights into the role of QLZDD in improving intestinal microbiota and discovers potential targets for the development of TS drugs. However, further studies are required to confirm the hypothesis.

## Data Availability

Accession to cite for these SRA data: PRJNA803670 Temporary Submission ID: SUB11037622 Release date: 2023-03-01 “Our SRA records will be accessible with the following link after the indicated release date: https://www.ncbi.nlm.nih.gov/sra/PRJNA803670”.
